# A106 COLONOSCOPY TEACHING: A SURVEY OF CANADIAN GASTROENTEROLOGY RESIDENTS

**DOI:** 10.1093/jcag/gwad061.106

**Published:** 2024-02-14

**Authors:** G Park, H Komeylian, A Kohansal, M Stewart

**Affiliations:** Dalhousie University, Halifax, NS, Canada; Dalhousie University, Halifax, NS, Canada; Dalhousie University, Halifax, NS, Canada; Dalhousie University, Halifax, NS, Canada

## Abstract

**Background:**

With the advancement in equipment and techniques, colonoscopy training continues to evolve. Programs such as the Skills Enhancement for Endoscopy (SEE) curriculum developed by the Canadian Association of Gastroenterology teach techniques such as loop reduction, patient turning, and water immersion aimed at improving adenoma detection, cecal intubation, and patient comfort. While there is vast literature focused on assessing learners’ endoscopic competencies, there is limited data pertaining to the training of specific colonoscopy techniques.

**Aims:**

To assess various colonoscopy techniques used to train gastroenterology residents in Canada.

**Methods:**

A survey of gastroenterology trainees was developed to assess specific aspects of their experience with colonoscopy education. This included questions on the frequency of position changes, use of pediatrics colonoscopes, water immersion, loops reduction, and SEE program initiatives. The survey was advertised at the 2023 Gastroenterology Residents-In-Training Course with a link to the survey e-mailed to all attendees. This survey has been re-distributed to the current gastroenterology resident cohort and therefore this abstract’s results are interim data pending further survey responses.

**Results:**

At the time of survey administration, there were 69 gastroenterology residents registered in Canada. Responses were received from 21(30%) residents; 5 in post graduate year (PGY) 4 and 16 in PGY5. One quarter (24%) of residents reported that colonoscopy teaching methods were uniform amongst faculty members and one third (33%) reported that more than half of their preceptors mentioned SEE program initiatives. Thirty-eight percent of residents reported that more than half of their preceptors suggested position change on insertion when the scope is advancing well on insertion. Majority of residents (90%) reported that water immersion during colonoscope insertion was recommended by most preceptors. Most residents (76%) reported that position change to help with loop reduction was suggested by more than half of their preceptors. Fifty-seven percent of residents reported that more than half of their preceptors commented on type of loop formation and reduction techniques. Once the cecum is reached, a minority of residents (29%) reported that more than half of preceptors recommended position change specifically to improve cecal visualization.

**Conclusions:**

Certain colonoscopy techniques such as turning patients, water immersion, and loop reduction can assist with adenoma detection rates and patient comfort. However, there exists considerable variability in colonoscopy techniques taught to Canadian gastroenterology residents. Increased standardization among colonoscopy technique education may benefit gastroenterology trainees.

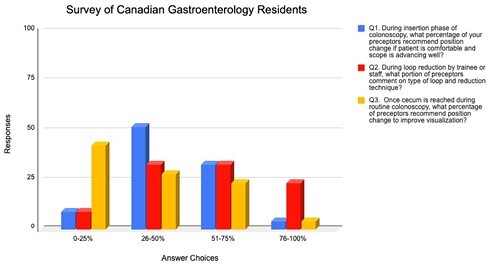

**Funding Agencies:**

None

